# The impacts of altruism levels on the job preferences of medical students: a cross-sectional study in China

**DOI:** 10.1186/s12909-023-04490-z

**Published:** 2023-07-28

**Authors:** Yue Zhang, Xing Lin, Xing Li, Youli Han

**Affiliations:** School of Public Health, Capital Medical University, No.10 Xitoutiao, Youanmenwai Street, Fengtai District, Beijing, 100069 China

**Keywords:** Altruism, Discrete choice experiment, Healthcare, Job preferences, Medical students, Recruitment and retention

## Abstract

**Background:**

Rational allocation of human resources for health is crucial for ensuring public welfare and equitable access to health services. Understanding medical students’ job preferences could help develop effective strategies for the recruitment and retention of the health workforce. Most studies explore the relationship between extrinsic incentives and job choices through discrete choice experiments (DCEs). Little attention has been paid to the influence of intrinsic altruism on job choice. This study aimed to explore the heterogeneous preferences of medical students with different levels of altruism regarding extrinsic job attributes.

**Methods:**

We conducted an online survey with 925 medical students from six hospitals in Beijing from July to September 2021. The survey combined job-choice scenarios through DCEs and a simulation of a laboratory experiment on medical decision-making behavior. Behavioral data were used to quantify altruism levels by estimating altruistic parameters based on a utility function. We fit mixed logit models to estimate the effects of altruism on job preference.

**Results:**

All attribute levels had the expected effect on job preferences, among which monthly income (importance weight was 30.46%, 95% CI 29.25%-31.67%) and work location (importance weight was 22.39%, 95% CI 21.14%–23.64%) were the most salient factors. The mean altruistic parameter was 0.84 (s.d. 0.19), indicating that medical students’ altruism was generally high. The subgroup analysis showed that individuals with higher altruism levels had a greater preference for non-financial incentives such as an excellent work environment, sufficient training and career development opportunities, and a light workload. The change in the rate of the uptake of a rural position by individuals with lower levels of altruism is sensitive to changes in financial incentives.

**Conclusions:**

Medical students’ altruism was generally high, and those with higher altruism paid more attention to non-financial incentives. This suggests that policymakers and hospital managers should further focus on nonfinancial incentives to better motivate altruistic physicians, in addition to appropriate economic incentive when designing recruitment and retention interventions. Medical school administrations could attach importance to the promotion of altruistic values in medical education.

**Supplementary Information:**

The online version contains supplementary material available at 10.1186/s12909-023-04490-z.

## Background

In recent decades, the world has experienced major health improvements. The universal health coverage (UHC) service index increased from a global average of 45 in 2000 to 66 in 2017 (WHO, 2019). However, health worker shortages and geographical disparities limit efforts to achieve UHC development goals by 2030. China has made significant progress over the past 10 years with regard to equal access to health care and financial protection [1, 2]. However, regional differences, particularly between urban and rural areas, continue to exist and are growing [3].

Urban–rural disparities have attracted more scholarly and governmental attention than regional discrepancies [4, 5]. By the end of 2021, the number of health professionals, practicing (assistant) physicians, and registered nurses per 1000 population in urban (rural) areas was 9.87 (6.27), 3.73 (2.42) and 4.58 (2.64), respectively (China Health Statistical Yearbook, 2022). Urban residents enjoy more access to health workers than rural residents [6]. Rural areas have sustained shortages of qualified health workers due to heavy workloads, poor infrastructure, and a lack of transportation [7]. Moreover, the dynamic nature of the healthcare workforce exacerbates retention difficulties. The allocation of more health workers to rural areas contributes to UHC, making the health system more equitable and efficient [2]. Therefore, the recruitment and retention of healthcare workers in rural areas have consistently ranked among the top concerns of healthcare systems worldwide [8]. An understanding of job preferences could provide useful information for the improvement of the recruitment and retention of health workforces and for the development of strategies designed for UHC.

Many studies have been conducted on employee turnover and retention and on the job preferences of graduate, undergraduate, and medical students. Scott et al. reviewed the reasons and characteristics of employee turnover and proposed the 5Cs strategies to improve retention, namely, communication, connection, collaboration, creating learning opportunities, crafting, and celebrating [9]. Wine et al. discussed predictors of turnover and concluded that training, supervision, pay, and different aspects of the job (e.g., professional development opportunities, under- or overscheduled) explained approximately 38% of the variance in turnover intention [10]. Engidaw et al. concluded that several factors affect graduate class medical students’ and other health professionals’ job preferences. These include allowance/salary, refreshment training, human resource management style, facility quality, and other individual characteristics (e.g., marital status, place of birth, and previous rural exposure) [11]. Liu et al. and Bao et al. explored the job preferences of Chinese medical undergraduates through DCEs and found that working in the city, a superior working environment, Bianzhi, and physical conflicts between doctors and patients were the most important non-monetary job characteristics [4, 12]. These findings contribute to the identification of the factors related to job preferences.

Ordinal approaches (e.g., DCEs, best–worst scaling, (BWS)) have been the main methods for accessing job preferences. Ordinal techniques offer certain advantages over cardinal tasks such as the standard gamble (SG) and time trade-off (TTO). While TTO and SG are designed to simulate the uncertainty of decision-making, the DCEs and BWS methods also incorporate situations in which the ideal alternative may not be an option and approach the complexity of decision-making in the real world. Moreover, cardinal tasks are considered particularly cognitively demanding, whereas ordinal tasks are easier to understand and complete [13]. A review of studies on DCEs and BSW in the field of health showed that DCEs and BWS generally provide similar preference estimates; however, DCEs have a higher response rate, stability, and continuity than BWS [14]. DCEs are more appropriate for quantifying job characteristics that influence health workers’ employment choices. DCEs are grounded in random utility theory and based on the assumptions of economic rationality and utility maximization. They permit an examination of the trade-offs between different factors and the rate of job choice uptake. DCEs may also be used to identify differences in preferences across groups [15]. Consequently, they have proven useful in determining diagnoses, treatments, access to services, and employment preferences of health personnel and students [16]. Although these studies have explored the influence of extrinsic incentives on medical students’ job preferences, they have not addressed the effect of intrinsic altruistic motivation.

In the principal-agent relationship between physicians and patients, altruism is an important intrinsic motivation conducive to patient welfare and implicit in ideas about medical professional values and attitudes [17]. Heterogeneity in altruism among medical students affects their preference for extrinsic incentives. Several studies have provided evidence that medical students with strong altruistic motivation are more likely to work in rural or underserved areas because the suboptimal work conditions that typically characterize those locations often require some level of self-sacrifice [18–23]. Some scholars have also identified substantial heterogeneity in altruism [24–26], suggesting the need to consider the complexity of altruistic motivations in any examination of job preferences among medical students. Conducting altruism analysis in job preference research can provide valuable evidence for improving medical students’ education and physicians’ invitation [18–23].

However, most studies on altruism have relied on self-reported data on the participants’ desire to help others, serve the poor, and provide services to vulnerable populations [19, 20, 22, 23, 27] or the allocation of money in the dictator game [8, 18, 21, 28]. Preferences for rural positions have also been identified using self-reported data on the likelihood that participants would choose to practice medicine in rural areas [18–23]. These approaches do not directly reflect the measure of altruism embedded in the physician–patient relationship, which is defined as the weight physicians place on the health benefits of patients in the utility function as opposed to the pursuit of their own interests [29], and fail to identify the breadth of considerations that go into choices made about financial and non-financial incentives.

DCEs have proven appropriate for studying extrinsic motivation with regard to job preferences. However, few scholars have examined the effects of intrinsic altruism. This absence could be attributed to difficulties intrinsic to quantifying medical altruism using field data [29]. Fortunately, recent economic experiments have provided a means to quantify physicians’ profits and patients’ health benefits. This study has shown ways to simulate the scenarios of physicians treating patients in controlled laboratories. Altruism could be estimated using the utility function for physicians [24] and the random utility model [25, 26].

This study aims to address this gap in the literature by combining an examination of altruism based on a measure of physicians’ profits and patients’ health benefits with DCEs. Therefore, we explored the effects of altruism on the influence of extrinsic incentives on medical students’ job preferences.

## Methods

### Study design

This cross-sectional study involved undergraduate and graduate students. The study was conducted between July and September 2021.

### Setting and sample

This study was conducted in Beijing, the capital city of China. Beijing has the most teaching hospitals in the country. Medical students from Beijing work in all regions of the country. Therefore, this research conducted in Beijing serves to inform strategies designed to address both the unequal distribution of local health workers and the allocation of the health workforce nationwide.

Our study included three subgroup analyses based on altruism level. G*Power version 3.1.9.7 [30] was used to set an effect size of 0.5, significance level of 0.05, power of 0.8, and sample size of the three groups was at least 42. Orme [31] and Johnson and Orme [32] recommended a sample of at least 200 respondents per group for subgroup analysis and the mean sample size of health-related DCEs for this type of research [16]. Therefore, we recruited a minimum of 600 participants and the calculated power was 1.

Cluster sampling was conducted at six teaching hospitals in Beijing. A total of 1834 eligible medical students were invited to participate in the study. The eligibility criteria included students in their third year or higher of medical school and postgraduates. Third-year cohorts were chosen because they had clinical internship experience and would soon enter the job market but were yet to make placement decisions [19, 33]. Eligible medical students were invited to participate in an online survey.

### Survey instrument

The survey instrument was a self-reported questionnaire consisting of six parts: preface, informed consent, basic personal information, a sequencing exercise with job characteristics, a lab-like online experiment on altruism, and DCEs choice tasks. Two blocks of questionnaires were randomly assigned. Respondents who answered that the same block faced the same order of choice.

A pilot survey of 150 eligible medical students was conducted to verify the validity of the questionnaire. The indirect statistical information on the design is presented in Supplementary Table 1. In the formal investigation, we sent the link and quick response (QR) code of the online questionnaire to hospital liaisons, who then forwarded it to eligible students. Restrictions, such as mandatory items, minimum time per page, and only one answer per ID, were used to control for missing data and the quality of answers. After the questionnaire was completed, the QR code of the WeChat group and contact information of the investigator were displayed. The respondents could scan the code or contact the investigator directly to join the group to get answers to relevant questions and the follow-up notification of the payment progress. The entire participation process took approximately 20 min to complete. Response data were automatically returned to our account on the WJX website.

A total of 925 eligible medical students signed informed consent forms and completed the survey, with a response rate of 50.4%. Among them, 50 were from non-sampled hospitals and were excluded from the total sample. Of the remaining 875 participants, approximately 15% (134) failed the internal consistency test and were excluded from the formal analysis. The respondents received the sum of the physicians’ profits from the laboratory-like online experiment of altruism plus a basic reward of 5 CNY. The aggregated patient health benefits generated by the physicians’ decisions were donated to the Red Cross Society of China. We recruited supervisors to ensure donation authenticity. They were paid an additional 50 CNY. Respondents received an average reward of 41 CNY. A total of 32,697 CNY was donated.

### Design of discrete choice experiment

The identification of attributes and assignment of their corresponding levels are the initial and key steps in DCEs, as they inform the subsequent formulation of alternatives and choice sets. The DCEs for medical students were classified according to the attributes that had the greatest influence on their job preferences. Financial incentives such as official income [34] and salary [35, 36] have always been considered the most important factors affecting job choices. However, non-financial incentives have also been shown to have effects that are sometimes equal to or exceed those of financial incentives. The salient nonfinancial factors that have appeared in previous studies include job location [12, 37], work conditions [38], supportive management [33], access to needed materials and space [39], and opportunities for career development [40–43]. Education for children [42], urban position reservation [36], workload [4, 37], and employment opportunities for partners or spouses [38] are also important considerations. Recognition by managers, peers, and the public has been shown to be an important motivating factor in healthcare settings [44–47]. Since this study was conducted in China, a summary of DCEs conducted for Chinese medical students found that monthly income, location, work environment, training and career development opportunities [12], and patient–doctor relationships [4] also had an important impact on job choices.

Based on a literature review of previous studies [48] and discussions with seven postgraduate medical students, senior health economists, and researchers familiar with DCEs, we identified six initial attributes and their corresponding levels: monthly income [12], work location [4, 12, 37], work environment [12], training and career development opportunities [4, 12, 40–43], workload [4, 37], and recognition from supervisors, peers, and the general public [44–47]. Based on data analysis of the pilot survey, we improved the levels of monthly income and workload. The attributes and levels are presented in Supplementary Table 2.

The six three-level attributes generated 729 (3^6^) hypothetical job scenarios and 265,356 ([729 × 728]/2) possible choice pairs. We used DCEs macros in SAS software version 9.4 to develop a D-optimal design that maximizes D-efficiency while considering orthogonality, level balance, and minimal overlap [49]. Given that we mainly focused on substitution effects between attributes, and the design plan of DCEs most frequently focused on main effects only [16], the interaction effects between attributes were not designed [50]. The design had 24 choice tasks, each with two alternatives, blocked into two sets of 12 choice tasks. A block of the choice set was randomly assigned to each respondent. Respondents first had to choose between two unlabelled job scenarios (e.g., Job A and Job B) and then choose whether they would engage in the selected job if it was available in real life (see Supplementary Table 3 for an example of a choice set). The two stages were combined for the analysis to consider the opt-out option [51]. We designed the experiment to more closely resemble real-life situations that respondents could face when they had the opportunity to choose non-participation. It also permitted maximization of the amount of information collected from each respondent [52, 53].

To test the internal consistency of respondents’ choices, we added a duplicate choice set of 13 to the original 12 choice sets for each block. One of the job scenarios was significantly superior to the others [34, 54]. The respondents were expected to choose the dominant alternative [37]. This step allowed us to identify responses that seemed random as opposed to representatives of thoughtful reflection [55].

### Lab-like online experiment

The use of economic experimental designs has been shown to be a robust method for measuring medical students’ altruism [24, 56]. In this study, we used an online approach to perform the measurements. Specifically, we designed a laboratory-like online experiment on physicians’ decision-making behavior under fee-for-service (FFS) conditions. Medical students played the role of physicians and decided on the quantity of medical services (*q*) to be provided to patients, which determined both their own profit *π*(*q*) (remuneration (*R*) - cost (*c*)) and the patients’ health benefit *B*(*q*). Physicians were compensated *p* for each unit of service they provided: remuneration *R*(*q*) = *pq*. *c*(*q*) was designed as a convex function, where *c*(*q*) = 0.1·*q*^2^ [57]. They were both measured in experimental currency units (Taler); *π*(*q*) and *B*(*q*) were paid to respondents and the charity organization at the exchange rate of two Talers for one CNY. Specifically, the experimental task was that physician *n*–according to the preset parameters of *R*(*q*), *c*(*q*), *π*(*q*), and *B*(*q*) corresponding to each quantity—chooses *q*$\in$ [0, 10] for nine different patients *j*$\in$ [1, 9] with three illnesses *k*$\in$ [*A*, *B*, *C*] and three levels of severity *l*$\in$ [*x*, *y*, *z*] (see Supplementary Table 4). The patients included in this study had a fictitious disease. They were assumed to be fully insured and passively accepted each service offered by their physicians. The patient order was predetermined as follows: *A*_*x*_, *B*_*y*_, *C*_*z*_, *B*_*x*_, *C*_*y*_, *A*_*z*_, *C*_*x*_, *A*_*y*_ and *B*_*z*_. Therefore, if *n* provides *q* for *j*, then *R*(*q*), *π*(*q*), *c*(*q*), and *B*(*q*) can be determined as follows:

1 $$B_{nj}\left(q\right)=B_k-\theta_k\left|q-q_l^\ast\right|$$

2 $$\pi_{nj}(q)\hspace{0.17em}=\hspace{0.17em}R_{nj}\left(q\right)\hspace{0.17em}-\hspace{0.17em}c\left(q\right)\hspace{0.17em}=\hspace{0.17em}p_k\cdot q\hspace{0.17em}-\hspace{0.17em}c\left(q\right)$$

*q*_l_^*^ is the quantity chosen to maximize *B*(*q*), and *q*_l_^*^ depends on *l*; the *q*_l_^*^ values of low (*x*), intermediate (*y*), and severe (*z*) are 3, 5, and 7, respectively. *B*_*k*_(*q*_l_^*^) is the maximum *B*(*q*), and *θ*_*k*_ is the marginal *B*(*q*) (a change in *B*(*q*) produced by an additional unit of *q*). They vary with *k*, *B*_*A*_(*q*_l_^*^) = 7; *B*_*B*_(*q*_l_^*^) = 10; *B*_*C*_(*q*_l_^*^) = 14; *θ*_*A*_ = *θ*_*B*_ = 1; and *θ*_*C*_ = 2. *p* is a fee per service, *p* > 0, *c*'(*q*) > 0, and *c*"(*q*) > 0. The *q*ˆ is the *q* chosen that maximized *π*(*q*). Because of the differences in *p* of *k* and the similarity between *π*(*q*ˆ) and *B*_*k*_(*q*_l_^*^), we set the *p* of *A*, *B* and *C* as 1.91, 2 and 2.1, respectively. *π*(*q*) was derived as *π*(*q*ˆ) at *q*ˆ = 10, π_*A*_(10) = 9.1, π_*B*_(10) = 10 and π_*C*_(10) = 11. Detailed descriptions of *B*(*q*) and π(*q*) are provided in Supplementary Table 5.

### Data analysis

#### Modelling of job preference

The random utility models provided a modelling framework for the DCEs data. The framework was based on the assumption that participants would choose the option with the highest utility. If individual *n* chooses alternative *j* from choice set *t*, then their utility can be expressed as

3 $$U_{njt}\hspace{0.17em}=\hspace{0.17em}\beta x_{njt}\hspace{0.17em}+\hspace{0.17em}\varepsilon_{njt},\;n\hspace{0.17em}=\hspace{0.17em}1,\;\dots,\;N;\;j\hspace{0.17em}=\hspace{0.17em}1,\;\dots,\;J;\;t\hspace{0.17em}=\hspace{0.17em}1,\;\dots,\;T,$$

where *x*_*njt*_ is the *k*-vector of observable attributes of alternative *j*, *β* is a vector of preference weights, and ε_*njt*_ is a random error term assumed to follow an independently and identically distributed type 1 extreme-value distribution, which is a function of unobserved job attributes and individual level variation in tastes.

Although the utility is not directly observable, the probability of choosing alternative *j* is

4 $$P(j|{X}_{nt})=\mathrm{exp}(\beta {x}_{njt})/\sum\nolimits_{k=1}^{J}\mathrm{exp}(\beta {x}_{nkt})$$

where *X*_*nt*_ is the vector of the attributes of all alternatives, *j* = 1, …, *J*.,

A conditional logit model (CLM) and mixed logit model (MLM) accommodating potential unobserved preference heterogeneity were used to estimate the utility function. The MLM is estimated by means of a maximum simulated likelihood estimation using 2000 Halton-draws. The log likelihood, Akaike information criterion (AIC), and Bayesian information criterion (BIC) were used for model comparison. All attributes were specified as having a random component [58], and all parameters were treated as normally distributed. The covariance matrix for the random coefficients is represented by a diagonal structure. Additionally, all attributes were coded using dummy variables except monthly income, which was coded as a continuous variable. For the opt-out option, all attributes were coded as 0 to avoid loss of data and power and to depict respondents’ preference not to accept either job [52, 59]. Based on the estimated coefficients, we also examined the respondents’ trade-offs between non-financial and financial attributes (willingness to pay, WTP), which indicated the amount of money a participant was willing to relinquish or receive in exchange for the corresponding improvement or deterioration of a certain attribute level [41]. WTP was estimated as the ratio of the mean estimated coefficient of a specific attribute level to the negative salary in the main effects of the MLM. Finally, we predicted the probability of engaging in a given job under simulated conditions; that is, the rate of acceptance of rural positions with the improvement of a certain attribute level or several attribute levels. The Stata command syntax used as an example of prediction can be found in Ryan et al. [60]. The WTP for attribute *x* and rate of uptake of the defined position are given by the following equations:

5 $$WTP(x)=-\frac{\partial U/\partial x}{\partial U/\partial salary}=-\frac{{\beta }_{x}}{{\beta }_{salary}}$$

6 $${P}_{njt}=\frac{{e}^{{\beta }^{\prime}{x}_{njt}}}{\sum_{k}{e}^{{\beta }^{\prime}{x}_{nkt}}}$$

### Quantification of altruism

Altruism is the degree to which utility-maximizing physicians attach importance to *B*(*q*) during the trade-off between *π*(*q*) and *B*(*q*). Altruism was quantified using the utility function of physicians based on behavioral data from the experimental part of the study. Therefore, physician *n* who chooses *q* to maximize utility, can be expressed as follows:

7 $$U_n\left(q\right)=\left(1\;-\;\alpha_n\right)\pi\left(q\right)+\alpha_nB\left(q\right)$$

*a*$\in$ [0, 1] is a measure of individual altruism; the larger the *a*, the higher the altruism; *a* = 0 represents a purely profit-maximizing physician and *a* = 1 represents a purely altruistic physician. Combined with Eqs. (1), (2) and (7), *a* may be calculated by using the first-order condition of the utility function. For *q* ≤ *q*_l_^*^, *a* = [2·0.1·*q* - *p*] / [2·0.1·*q* - *p* + *θ*_*k*_]; *q* ≥ *q*_l_^*^, *a* = [2·0.1·*q* - *p*] / [2·0.1·*q* - *p* - *θ*_*k*_]. The expression of the calculated *a* is shown in Supplementary Table 6. To ensure that *a* = 1 when physicians select *q*_l_^*^, we performed a standardization; *a* divided by the *a*_*opt*_ corresponding to the *q*_l_^*^ of the same patient. We calculated the individual *a* only for respondents who chose half or more of the Pareto-efficient *q*, which is the quantity choice in the range between *q*_l_^*^ and *q*ˆ. The individual *a* was the mean of the *a*’s of nine different patients.

We also adhered to Godager and Wiesen’s recommendation [25] and tested the robustness of the *a* estimated based on the above utility function (7) using the estimated marginal rate of substitution (MRS) between *B*(*q*) and *π*(*q*) from the MLM. The degree of altruism was determined by comparing the MRS and the 1. The larger the MRS, the higher the altruism. If the MRS score was greater than 1, the physicians assigned greater weight to *B*(*q*) than to *π*(*q*). If the MRS was equal to 1, it would mean that the physicians were equally important to *B*(*q*) and *π*(*q*). Finally, if the MRS score was less than 1, the physicians granted greater weight to *π*(*q*) than to *B*(*q*).

### The effects of altruism on job preference

This two-step analysis examined the effect of altruism as an intrinsic motivation for the influence of job characteristics on job preference. First, we incorporated the interaction term between *a* and each attribute level into the MLM. The statistical significance and symbols of the coefficients of the interaction term were used to judge whether altruism had an effect and the direction of such an effect. In cases where the interaction effects model showed significant differences between groups, separate stratified models (subgroup analyses) were used to describe the preferences of each group.

All statistical analyses were performed using Stata version 16 (StataCorp LP, College Station, Texas, USA).

## Results

### Descriptive statistics of demographics

We analyzed the demographics of the respondents who passed and failed the internal consistency test using a chi-squared test. The results showed no differences in distribution between the two groups. Specifically, 741 respondents with an average age of 24.0 years passed the internal consistency test. Women accounted for approximately 60% of the sample, and most were from the city (58%) and were only children (61%). The demographic details are presented in Table 1.

**Table 1:** Descriptive statistics for medical students

Demographics	All (*n* = 875, %)	Analysis Sample (*n* = 741, %)	Excluded Sample (*n* = 134, %)	χ^2^	*p*-value
**Age**	24.1 (± 2.9)	24.0 (± 2.7)	24.5 (± 3.5)		
**Gender**					
Women	529 (60.5)	442 (59.7)	87 (64.9)	1.321	0.250
Men	346 (39.5)	299 (40.3)	47 (35.1)		
**Birthplace**					
Township or village	228 (26.1)	189 (25.5)	39 (29.1)	1.040	0.594
County	143 (16.3)	120 (16.2)	23 (17.2)		
City	504 (57.6)	432 (58.3)	72 (53.7)		
**Single child**					
No	345 (39.4)	293 (39.5)	52 (38.8)	0.026	0.873
Yes	530 (60.6)	448 (60.5)	82 (61.2)		
**Amount of money needed for monthly expenses (CNY)**					
< 800	25 (2.9)	21 (2.8)	4 (3.0)	2.599	0.458
800 - 1499	262 (29.9)	215 (29.0)	47 (35.1)		
1500 - 2499	390 (44.6)	338 (45.6)	52 (38.8)		
= 2500	198 (22.6)	167 (22.5)	31 (23.1)		
**Annual family income (CNY)**					
< 30,000	94 (10.7)	78 (10.5)	16 (11.9)	3.459	0.484
30,000 - 49,999	137 (15.7)	117 (15.8)	20 (14.9)		
50,000 - 69,999	121 (13.8)	106 (14.3)	15 (11.2)		
70,000 - 99,999	130 (14.9)	104 (14.0)	26 (19.4)		
= 100,000	393 (44.9)	336 (45.3)	57 (42.5)		
**Career planning**					
Engage in health-related work	380 (43.4)	313 (42.2)	67 (50)	6.385	0.094
Engage in non-health related work	8 (0.9)	5 (0.7)	3 (2.2)		
Continue education	479 (54.7)	416 (56.1)	63 (47.0)		
Others	8 (0.9)	7 (0.9)	1 (0.8)		

### Ranking of job characteristics

Prior to the DCEs, respondents ranked the importance of the factors that influenced their job choices. The results showed that monthly income and work location were the most important factors for participants’ job choices, followed by work environment, training, and career development opportunities. Professional recognition and workload were less important (Supplementary Fig. 1). This outcome was consistent with the strength of the relative importance of job characteristics based on the DCEs data, suggesting that the results derived from the DCEs had good internal predictive validity.

### Estimation of job preference modelling

We further quantified and verified the importance of these factors by using DCEs. By fitting the MLM and CLM to the DCEs data, the AIC and BIC values suggested that the MLM was preferable to the CLM. The estimates of the MLM are reported in Table 2, while those of the CLM are presented in Supplementary Table 7. We found that the coefficients for each attribute level were statistically significant, showing that all attributes had an impact on respondents’ job preferences. Among them, monthly income (30.46%) and work location (22.39%) were of the greatest importance. All coefficients are positive, indicating that respondents obtain more utility from a higher reference level than from a lower reference level. According to the preference weight reflected by the size of the coefficients, non-financial incentives, such as work location city ($\widehat{\beta }$ = 2.493), excellent work environment ($\widehat{\beta }$ = 1.751), and sufficient training and career development opportunities ($\widehat{\beta }$ = 1.534), were highly valued, while workload and professional recognition were relatively less important. Given that the standard deviations of most coefficients are statistically significant, the preferences for different attribute levels are heterogeneous among the samples. The details are presented in Table 2. Moreover, the MLM estimates based on the DCEs data of the full sample of 875 respondents were used for the sensitivity analysis, which showed no substantial difference from the estimates of the 741 respondents who passed the internal conformance test (see Supplementary Table 8). Although we used the independent random parameter MLM as our main model, we estimated a correlated specification for the sensitivity analyses. Sensitivity analyses show that a correlated specification provides very similar estimates and makes virtually no difference to the main conclusion.

**Table 2:** Estimation of mixed logit model for job preferences

Attribute level	Coef (SE)	SD (SE)	Importance (%, 95% CI)
ASC (opt-out)	9.621*** (0.301)	2.467*** (0.135)	
**Monthly income**	0.0005652*** (0.0000181)	0.0001401*** (0.0000146)	30.46 (29.25, 31.67)
**Work location: village or township (ref)**			
County	1.048*** (0.080)	0.787*** (0.108)	
City	2.493*** (0.104)	1.367*** (0.090)	22.39 (21.14, 23.64)
**Work environment: poor (ref)**			
Common	1.319*** (0.082)	0.757*** (0.105)	
Excellent	1.751*** (0.084)	0.520*** (0.141)	15.73 (14.53, 16.93)
**Training and career development opportunities: insufficient (ref)**			
General	0.518***(0.071)	0.214 (0.166)	
Sufficient	1.534*** (0.090)	1.196*** (0.085)	13.78 (12.53, 15.02)
**Workload: 60 h/week (ref)**			
50 h/week	0.381*** (0.067)	0.014 (0.221)	
40 h/week	0.880*** (0.072)	0.778*** (0.096)	7.90 (6.70, 9.09)
**Professional recognition: low (ref)**			
Normal	0.815*** (0.072)	0.090 (0.159)	
High	1.085*** (0.079)	0.798*** (0.096)	9.74 (8.53, 10.96)
N	741		
Observation	26,559		
Log likelihood	- 6016.956		
LR χ^2^	2594.57		
Prob > χ^2^	< 0.0001		
AIC	12,081.91		
BIC	12,278.40		

Since monthly income was treated as a continuous variable, its estimated coefficient was less than 0.001

*Coef* mean estimated coefficient, *SE* standard error, *SD* standard deviation, indicating preference heterogeneity, *CI* confidence interval (delta method), *AIC* Akaike Information Criterion, *BIC* Bayesian Information Criterion

^***^p < 0.001

**Fig. 1:**
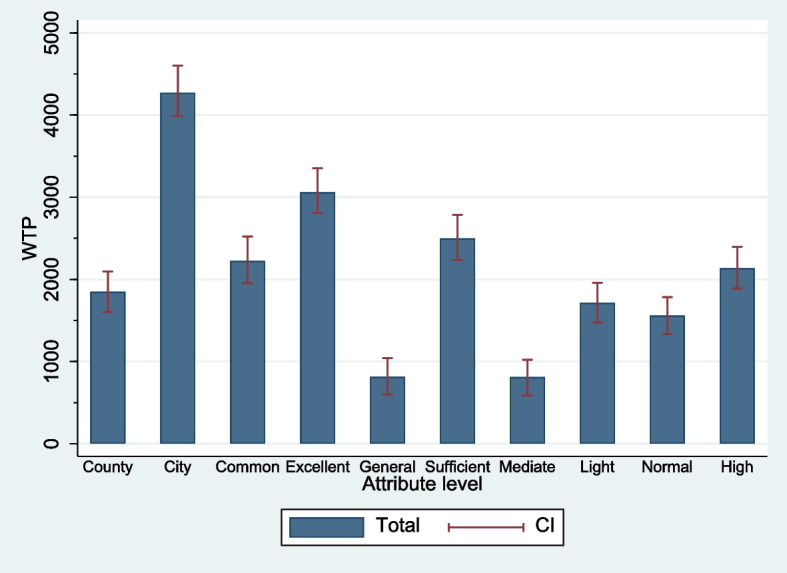
Willingness to pay for non-financial attribute levels. County and City are the work locations; Common and Excellent are the levels of work environment; General and Sufficient are the levels of training and career development opportunities; Average (50 h/week) and Light (40 h/week) are the levels of workload; Normal and High are the levels of professional recognition. CI: The confidence intervals of WTP (Krinsky-Robb parametric bootstrap)

Based on the above estimates, we transform the relative importance of different non-financial attributes into monetary values with more policy implications by calculating the marginal WTP. The results showed that respondents were willing to relinquish an average of 4410 CNY per month for an urban job compared to working in a rural or township area. They were also willing to devote 3099 CNY per month to improving their work environment from poor to excellent. They were willing to earn an average of 2714 CNY less per month to secure a job with sufficient opportunities for training and career development. Moreover, a high level of professional recognition amounted to 1920 CNY per month. However, changing workloads from 60 to 40 h a week only resulted in a cost of approximately 1556 CNY per month (see Fig. 1).

Finally, we predict the influence of changes in job characteristics on the rate of uptake of a simulated job. A comparison of rural and urban positions revealed that respondents at baseline were only 7.6% more likely to accept rural positions. With an improvement in the attribute levels, the probability gradually increased. The focus on a single incentive improvement demonstrated that a monthly income of 9000 CNY increased the probability of rural position acceptance to 31.1%. Excellent work environments matched this effect (32.3%). However, a monthly income of 12,000 CNY had the greatest effect (71.1%). When incentives were combined, excellent work environment and sufficient training and career development opportunities increased the uptake rate of rural positions to 68.8%, the effect of 9000 CNY per month coupled with excellent work environment increased slightly (72.2%), and the probability of a monthly income of 9000 CNY together with excellent work environment and sufficient training and career development opportunities increased to 92.3% (Supplementary Fig. 2).

### Estimation of altruistic preference

**Fig. 2:**
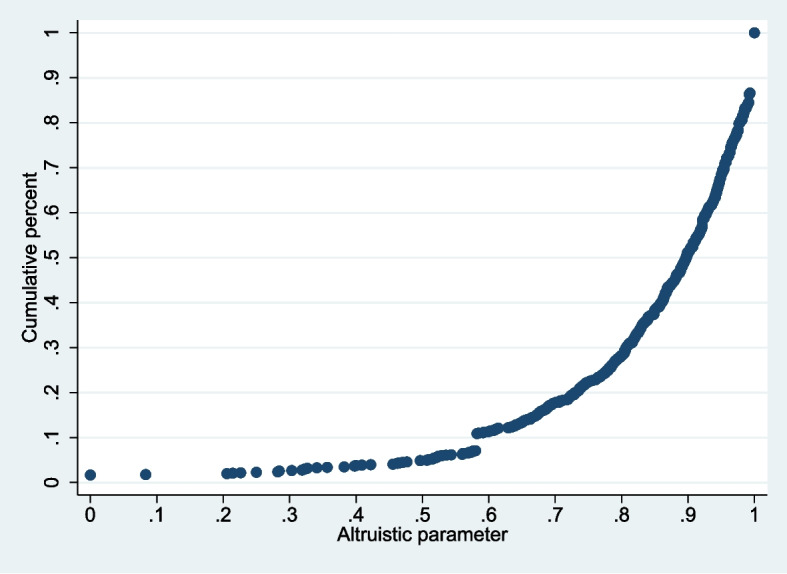
Cumulative frequency distribution graph of the altruistic parameter (*a*) at individual level

We quantified physician altruism through *a* and MRS. A total of 820 respondents chose half or more of the Pareto-efficient *q*. On average they attached a positive weight to *B*(*q*). A total of 14 of them chose *q*ˆ; the *a* was 0. A total of 83 participants always chose *q*_l_^*^; the *a* was 1. The mean value of *a* was 0.84 (s.d. 0.19). The cumulative frequency distribution graph of *a* shown in Fig. 2 illustrates substantial heterogeneity in *a*. The *a* was larger than 0.5 for approximately 95% of the respondents, and half of the respondents had an *a* above 0.9. The overall MRS was 1.11 (Supplementary Table 9), and the correlation analysis between the MRS and *a* showed a significant positive correlation (Spearman’s *ρ* = 0.839, *p* < 0.001).

### Estimation of the effect of altruism on job preference

**Fig. 3:**
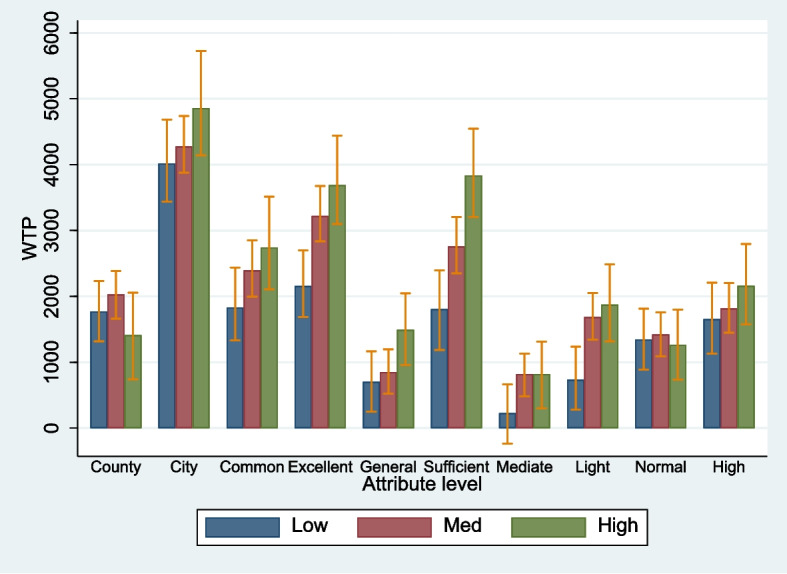
Willingness to pay of subgroups with different levels of altruism. The meaning of the label on the horizontal axis is the same as that on the horizontal axis in Fig. 1. Low: low-altruism group; Med: medium-altruism group; High: high-altruism group

Based on the effective verification of the altruism estimate, we further analyzed the differences in job preferences among physicians with different levels of altruism. The MLM that incorporated the interactions between *a* and each attribute level yielded a negative coefficient for the interaction between *a* and monthly income. This proves that the effect of monthly income on job preference is relatively small for respondents with higher altruism. The coefficients of the interactions between *a* and excellent work environment ($\widehat{\beta }$ = 1.152), sufficient training and career development opportunities ($\widehat{\beta }$ = 1.543), and light workload ($\widehat{\beta }$ = 1.233) were significantly positive. This demonstrates that respondents with higher altruism have a greater degree of preference for these factors in their job choices (Table 3). We further divided the respondents into three groups (low, medium, and high) based on the quartiles of altruism (0.78, 0.97), and then grouped them to fit the MLM. The coefficient of each attribute level for the three subgroups was significantly positive (0.0005811 ≤ $\widehat{\beta }$ ≤ 2.823), with that of monthly income the smallest in the high-altruism group and the largest in the low-altruism group (supplementary Tables 10–12). The relative importance of non-financial attribute levels was compared using the WTP (Fig. 3 and Supplementary Tables 10–12). The results show that the WTP of respondents in the high-altruism group was higher than that of respondents in the medium- and low-altruism groups. However, there were no significant differences in the rates of uptake of rural positions at baseline among the three groups. The change in this probability under the simulated conditions showed that the improvement in non-financial attribute levels and their corresponding combinations increased it the most in the high- and medium-altruism groups, whereas the improvement in monthly income increased it the most in the low-altruism group. This finding indicates that respondents in the high-altruism group were more sensitive to improvements in non-financial attribute levels, while respondents in the low-altruism group were more sensitive to improvements in financial incentives (Table 4).

**Table 3:** Estimation of mixed logit model of attribute level with altruism interaction

Attribute levels	Coef (SE)	SD (SE)
ASC (opt-out)	7.980*** (1.075)	2.541*** (0.134)
**Monthly income**	0.0006364*** (0.0000689)	0.0001256*** (0.0000147)
**Work location: village or township (ref)**		
County	1.216** (0.367)	0.809*** (0.116)
City	2.437*** (0.445)	1.443*** (0.097)
**Work environment: poor (ref)**		
Common	1.019** (0.363)	0.802*** (0.101)
Excellent	0.810* (0.350)	0.426* (0.206)
**Training and career development opportunities: insufficient (ref)**		
General	0.119 (0.323)	0.073 (0.195)
Sufficient	0.280 (0.408)	1.135*** (0.086)
**Workload: 60 h/week (ref)**		
50 h/week	- 0.385 (0.313)	0.070 (0.233)
40 h/week	- 0.173 (0.340)	0.675*** (0.100)
**Professional recognition: low (ref)**		
Normal	1.201*** (0.331)	0.147 (0.181)
High	0.952** (0.363)	0.809*** (0.099)
Altruism*ASC (opt-out)	2.291* (1.246)	
Altruism*Monthly income	- 0.0000659 (0.0000788)	
Altruism*County	- 0.156 (0.428)	
Altruism*City	0.107 (0.515)	
Altruism*Common work environment	0.382 (0.425)	
Altruism*Excellent work environment	1.152** (0.410)	
Altruism*General training and career development opportunities	0.494 (0.379)	
Altruism*Sufficient training and career development opportunities	1.543** (0.477)	
Altruism*50 h/week	0.905* (0.367)	
Altruism*40 h/week	1.233** (0.396)	
Altruism*Normal professional recognition	- 0.470 (0.386)	
Altruism*High professional recognition	0.158 (0.422)	
N	695	
Observation	24,906	
Log likelihood	- 5576.2514	
LR χ^2^	2449.23	
Prob > χ^2^	< 0.0001	

Since monthly income was treated as a continuous variable, its estimated coefficient was less than 0.001

*Coef* mean estimated coefficient, *SE* standard error, *SD* standard deviation, indicating preference heterogeneity

^***^p < 0.001, ^**^p < 0.01, ^*^p < 0.1

**Table 4:** Prediction of the rate of uptake of rural position under simulated incentive packages

Low-altruism group		Medium-altruism group		High-altruism group	
Incentive packages	Uptake rate	Incentive packages	Uptake rate	Incentive packages	Uptake rate
Baseline	7.3%	Baseline	7.3%	Baseline	5.6%
40	11.1%	40	17.7%	40	15.0%
HPR	18.3%	HPR	18.9%	HPR	17.3%
STC	19.8%	STC	28.9%	9000	25.4%
EWE	23.5%	9000	31.9%	EWE	33.7%
9000	34.4%	EWE	34.8%	STC	35.6%
EWE + HPR	46.7%	EWE + HPR	61.1%	EWE + HPR	64.1%
EWE + STC	49.2%	9000 + STC	70.6%	12,000	66.0%
9000 + STC	62.3%	EWE + STC	73.3%	9000 + EWE	74.4%
9000 + EWE	67.3%	12,000	73.5%	9000 + STC	76.0%
EWE + STC + HPR	73.4%	9000 + EWE	76.0%	EWE + STC	82.5%
12,000	77.8%	EWE + STC + HPR	88.9%	EWE + STC + HPR	94.3%
9000 + EWE + STC	86.6%	9000 + EWE + STC	94.2%	9000 + EWE + STC	96.4%

Baseline: All incentives are the poorest (reference levels)

*40* 40 h/week, *HPR* High professional recognition, *STC* Sufficient training and career development opportunities, *EWE* Excellent work environment, *9000* 9000 CNY/month, *12,000* 12,000 CNY/month

## Discussion and conclusions

### Key results and interpretation

Among the nonfinancial attribute levels, work location yielded the largest preference. This was consistent with findings from previous studies [12, 37]. This result may be related to stereotypes regarding healthcare work in rural areas, such as lack of infrastructure, heavy workloads, poor hospital management, and isolation [7, 61]. However, the factors hindering students from different backgrounds from accepting rural positions also differ. Azer et al. [62] reported that, for rural students, the main barriers were spouse/partner needs and school availability for their children, whereas for urban students, the barriers were personal factors, education opportunities, social/cultural facilities, and the need for frequent travel.

Work environment and conditions also proved to be important for a variety of reasons related to infrastructure. These findings are consistent with those of studies conducted in Ghana [33], the UK [38], and Zambia [39]; good work conditions and housing availability had the greatest impact on job preferences. Additionally, a study from China showed that unsafe work environments can have a significant negative impact on medical students’ choices [4].

DCEs conducted in Tanzania [41], Uganda [43], India [42], and Indonesia [40] all revealed that future educational tuition is strongly associated with job choices. Hou et al. [63] conducted a nationwide survey in China and found that good career prospects rank first in terms of influencing job choices. Although professional recognition is strongly recommended by the WHO [46] for the overall ranking of recruitment and retention interventions in rural areas, it was not found to be a major concern in DCEs for medical students. We only found this in studies involving healthcare workers. A DCE study conducted by Saran et al. [47] on community health workers in Western Kenya found that improving community members’ recognition of their contributions significantly affected their motivation and permanence in their positions. Another DCE of community health volunteers in Kenya found that they preferred jobs that provided recognition at the community level [44]. Finally, the effect of workload was not as strong as that of other attributes. This finding is consistent with those reported by Bao and Huang [4] and Rao et al. [42].

Financial incentives are considered to be one of the most effective interventions for improving the uptake rates of rural positions. In this study, the uptake rate of the designed rural position at baseline was only 7.6%, a rate similar to the 8.7% of students who wanted to work in rural areas in a survey conducted by Syahmar et al. [64] in Indonesia. A survey conducted by Liu et al. [65] in Shaanxi, China, showed that only 7.4% of rural-oriented medical students with tuition waivers expressed intentions to fulfill their six-year contract to work in rural township hospitals after graduation; only 1.3% intended to remain in rural areas after their contract expired. However, increased monthly income is a powerful incentive to accept rural positions. This outcome is consistent with DCEs conducted in Vietnam [34], Cameroon [36], and Laos [35], where salary was the most important factor affecting medical students’ job preferences. Financial incentives have also been found to be associated with job satisfaction among rural-oriented medical students [66]. This effect was more pronounced in the low-altruism group.

The altruism parameter *a* of 0.84 (s.d., 0.19) in our study was higher than the 0.75 (s.d., 0.26) in Brosig-Koch et al. [24]. This could be attributed to the fact that the *a* in their study was the mean the *a* for patients in the pure and mixed payment schemes, whereas the *a* in this study, it was only the mean the *a* for patients in the FFS. The coefficients and their corresponding standard deviations imply that 94% of the respondents yield a positive marginal utility of *B*(*q*), whereas 89% have a positive marginal utility of *π*(*q*). The overall MRS was 1.11 > 1, indicating that physicians attached greater weight to *B*(*q*) than to *π*(*q*). It was lower than the 1.84 in Godager and Wiesen’s [25] and the 1.592 and 2.180 in Wang et al.’s [26] studies. However, the percentage of respondents with MRS values greater than 1 (49.4%) was higher than the 44% in Godager and Wiesen’s study [25]. The cumulative frequency distribution graph of MRS showed substantial heterogeneity (Supplementary Fig. 3).

There have been a few investigative and experimental studies that have served to predict the effect of altruism on the possible acceptance of rural work. Surveys conducted in Rwanda [22], Ghana [19], Argentina [20], and Asia and Africa [23] showed that medical students with high intrinsic altruistic motivation have a greater self-reported likelihood of accepting work in rural areas. Similar results were found in dictator game experiments conducted by Lagarde and Blaauw [18] in South Africa and by Li [21] in the United States. Gyorffy et al. [27] also found that altruistic motivation was the most significant career choice factor. The absence of altruistic motivation is the main risk factor for burnout. This study did not find significant differences in the rate of uptake of a rural position at baseline among the different subgroups of altruism. However, the high-altruism group was relatively more sensitive to improvements in attribute levels, and the corresponding rate in the high-altruism group that selected rural positions was also higher. Therefore, it is important to encourage medical students to become altruistic physicians in order to improve healthcare accessibility. With the development of artificial intelligence (AI) in medical education, humanistic education will be emphasized more because it is difficult to replace with technology [67]. Education that promotes altruism will help guide medical students and future physicians to better utilize AI for active learning, thereby providing better patient health services.

### Conclusions

This study contributes significantly to the literature on medical students’ job preferences and altruism. The results of this study demonstrate that the effect of extrinsic incentives on job preferences differs among medical students with different levels of altruism. Therefore, policymakers and hospital managers should include targeted interventions for individuals with different altruism levels, and further focus on nonfinancial incentives to better motivate altruistic physicians, in addition to appropriate economic incentive when designing recruitment and retention interventions. Medical school administrations could also focus on the enrolment of students from rural backgrounds, the inclusion of courses that address health issues specific to rural populations, clinical rotations and practices in rural areas, and more attention to the promotion of altruistic values in medical education. Efforts have also been pursued through social media. For example, a public relations campaign focusing on the social value of rural work and the appeal of altruism could be developed to enhance recognition of professionals employed in rural areas.

### Limitations

This study has several limitations. First, we conducted this study online due to COVID-19 restrictions. Although it has been reported that participants behave similarly in online and laboratory experiments [68], the investigation in this study included more comprehensive content and complex experimental tasks than previous studies. Consequently, the effect of online experiments in this study might have been affected compared to face-to-face on-site surveys. Second, respondents may have a recall bias for medical decision-making and job preferences. We tried to deal with these issues through a design that simulates real-world situations, large-sample surveys, and subgroup analysis. Third, the study was conducted in Beijing, China. Therefore, the applicability of these results to other settings is limited. Finally, this study adopted the same attribute levels for the DCEs of medical undergraduates and postgraduates. Although some scholars have adopted the same attribute levels for medical students and physicians [34] and nursing students and nurses [69] in their studies, respondents at different stages of their careers could differ in their prioritization of attributes that impact their job preferences. In the future, further consideration should be given to the use of different DCEs attribute levels to conduct field surveys among medical undergraduates and postgraduates nationwide as a means by which to extend the findings from this study.

## Supplementary Information

**Additional file 1.**

## Data Availability

The data for this study are available from the corresponding author, YLH, upon reasonable request.

## Abbreviations

UHC: Universal health coverage
SG: Standard gamble
TTO: Time trade-off
BWS: Best–worst scaling
DCEs: Discrete choice experiments
QR: Quick response
CNY: Chinese Yuan
FFS: Fee-for-service
CLM: Conditional logit model
MLM: Mixed logit model
AIC: Akaike information criterion
BIC: Bayesian information criterion
WTP: Willingness to pay
MRS: Marginal rate of substitution
AI: Artificial intelligence
