# Supplementary material for: The impacts of altruism levels on the job preferences of medical students: a cross-sectional study in China
Source: BMC Med Educ. 2023 Jul 28;23:538. doi: 10.1186/s12909-023-04490-z (PMC10375683; doi:10.1186/s12909-023-04490-z)
Supplement: Supplementary file 1 — Additional file 1. [file 12909_2023_4490_MOESM1_ESM.docx]

# Appendix

**Table 1** The indirect statistical information for choice sets design

| Design | 32 |
| --- | --- |
| Choice Sets | 24 |
| Alternatives | 2 |
| Parameters | 12 |
| Maximum Parameters | 24 |
| D-Efficiency | 17.8590 |
| Relative D-Efficiency | 74.4125 |
| D-Error | 0.0560 |
| 1 / Choice Sets | 0.0417 |

**Table 2** Attributes and levels for DCE

| Attributes | Definition | Levels |
| --- | --- | --- |
| Monthly income | Monthly income refers to pre-tax income, including basic salary, bonuses, and various benefits. | 6000 CNY/month |
|  |  | 9000 CNY/month |
|  |  | 12,000 CNY/month |
| Work location | Work location refers to work in various medical and health institutions in different regions. | Village or township |
|  |  | County |
|  |  | City |
| Work environment | Work environment includes access to needed equipment and supplies, such as medications, and support from management, amenities, and positive interpersonal relationships. | Poor |
|  |  | Common |
|  |  | Excellent |
| Training and career development opportunities | Training and career development opportunities refer to professional title assessment, career promotion, short-term training, and long-term continuing education. | Insufficient |
|  |  | General |
|  |  | Sufficient |
| Workload | Workload includes daily workload, overtime, on-duty hours, and night shifts. | 60 h/week |
|  |  | 50 h/week |
|  |  | 40 h/week |
| Professional recognition | Professional recognition refers to the degree of recognition of the valued placed on their jobs by the general public, patients, and families. | Low |
|  |  | Normal |
|  |  | High |

**Table 3** An example of one DCE choice set

| Attributes | Job A | Job B |
| --- | --- | --- |
| Monthly income | 12,000 CNY | 6000 CNY |
| Work location | Village or township | City |
| Work environment | Excellent | Poor |
| Training and career development opportunities | Insufficient | General |
| Workload | 50 h/week | 40 h/week |
| Professional recognition | High | Normal |
| Job preference |  |  |
| Job preference in real-life situation |  Yes | |
|  |  No | |

**Table 4** An example of one medical decision-making scenario

| Medical service (Patient: *B_y_*) | Quantity | Payment | Cost | Profit | Patient benefit |
| --- | --- | --- | --- | --- | --- |
| None | 0 | 0 | 0 | 0 | 5 |
| Service 1 | 1 | 2 | 0.1 | 1.9 | 6 |
| Service 1, Service 2 | 2 | 4 | 0.4 | 3.6 | 7 |
| Service 1, …, Service 3 | 3 | 6 | 0.9 | 5.1 | 8 |
| Service 1, …, Service 4 | 4 | 8 | 1.6 | 6.4 | 9 |
| Service 1, …, Service 5 | 5 | 10 | 2.5 | 7.5 | 10 |
| Service 1, …, Service 6 | 6 | 12 | 3.6 | 8.4 | 9 |
| Service 1, …, Service 7 | 7 | 14 | 4.9 | 9.1 | 8 |
| Service 1, …, Service 8 | 8 | 16 | 6.4 | 9.6 | 7 |
| Service 1, …, Service 9 | 9 | 18 | 8.1 | 9.9 | 6 |
| Service 1, …, Service 10 | 10 | 20 | 10.0 | 10.0 | 5 |
| Please fill in the quantity of medical services you would like to provide (0–10): _____________ | | | | | |

**Table 5** The Design of *B*(*q*) and *π*(*q*)

| Patient | *θ_k_* | *q_l_*^*^ | *B_k_*(*q_l_*^*^) | *p* | *R* | *q*ˆ | *π*(*q*ˆ) |
| --- | --- | --- | --- | --- | --- | --- | --- |
| *A_x_* | 1 | 3 | 7 | 1.91 | 1.91*q* | 10 | 9.10 |
| *A_y_* | 1 | 5 | 7 | 1.91 | 1.91*q* | 10 | 9.10 |
| *A_z_* | 1 | 7 | 7 | 1.91 | 1.91*q* | 10 | 9.10 |
| *B_x_* | 1 | 3 | 10 | 2.00 | 2.00*q* | 10 | 10.00 |
| *B_y_* | 1 | 5 | 10 | 2.00 | 2.00*q* | 10 | 10.00 |
| *B_z_* | 1 | 7 | 10 | 2.00 | 2.00*q* | 10 | 10.00 |
| *C_x_* | 2 | 3 | 14 | 2.10 | 2.10*q* | 10 | 11.00 |
| *C_y_* | 2 | 5 | 14 | 2.10 | 2.10*q* | 10 | 11.00 |
| *C_z_* | 2 | 7 | 14 | 2.10 | 2.10*q* | 10 | 11.00 |

**Table 6** Unstandardized *α*

| Patient | *θ_k_* | *p* | *q* ≤ *q*^*^ | *q* ≥ *q*^*^ |
| --- | --- | --- | --- | --- |
| *A_x_* | 1 | 1.91 | (0.2*q* − 1.91)/(0.2*q* − 0.91) | (0.2*q* − 1.91)/(0.2*q* − 2.91) |
| *A_y_* | 1 | 1.91 | (0.2*q* − 1.91)/(0.2*q* − 0.91) | (0.2*q* − 1.91)/(0.2*q* − 2.91) |
| *A_z_* | 1 | 1.91 | (0.2*q* − 1.91)/(0.2*q* − 0.91) | (0.2*q* − 1.91)/(0.2*q* − 2.91) |
| *B_x_* | 1 | 2 | (0.2*q* − 2)/(0.2*q* − 1) | (0.2*q* − 2)/(0.2*q* − 3) |
| *B_y_* | 1 | 2 | (0.2*q* − 2)/(0.2*q* − 1) | (0.2*q* − 2)/(0.2*q* − 3) |
| *B_z_* | 1 | 2 | (0.2*q* − 2)/(0.2*q* − 1) | (0.2*q* − 2)/(0.2*q* − 3) |
| *C_x_* | 2 | 2.1 | (0.2*q* − 2.1)/(0.2*q* − 0.1) | (0.2*q* − 2)/(0.2*q* − 4.1) |
| *C_y_* | 2 | 2.1 | (0.2*q* − 2.1)/(0.2*q* − 0.1) | (0.2*q* − 2)/(0.2*q* − 4.1) |
| *C_z_* | 2 | 2.1 | (0.2*q* − 2.1)/(0.2*q* − 0.1) | (0.2*q* − 2)/(0.2*q* − 4.1) |

**Fig. 1.** The importance of job attributes. Labels 1–6 on the horizontal axis indicate the order of importance from highest to lowest.

**Table 7** Estimation of conditional logit model for job preferences

| Attribute level | Coef (SE) | Importance (%, 95% CI) |
| --- | --- | --- |
| ASC (opt-out) | 6.637*** (0.145) |  |
| **Monthly income** | 0.0003753***(9.09e-06) | 30.62 (29.25, 31.71) |
| **Work location: village or township (ref)** |  |  |
| county | 0.767*** (0.052) |  |
| city | 1.592*** (0.052) | 21.65 (20.55, 22.75) |
| **Work environment: poor (ref)** |  |  |
| common | 0.758*** (0.049) |  |
| excellent | 1.118*** (0.048) | 15.20 (14.08, 16.33) |
| **Training and career development opportunities: insufficient (ref)** |  |  |
| general | 0.440***(0.051) |  |
| sufficient | 1.048*** (0.052) | 14.25 (13.11, 15.39) |
| **Workload: 60 h/week (ref)** |  |  |
| 50 h/week | 0.252*** (0.048) |  |
| 40 h/week | 0.505*** (0.047) | 6.87 (5.66, 8.08) |
| **Professional recognition: low (ref)** |  |  |
| normal | 0.583*** (0.049) |  |
| high | 0.839*** (0.050) | 11.40 (10.19, 12.62) |
| N | 741 | |
| Observation | 26559 | |
| Log likelihood | −7314.2406 | |
| LR χ^2^ | 4823.55 | |
| Prob > χ^2^ | < 0.0001 | |
| AIC | 14652.48 | |
| BIC | 14750.73 | |

^***^*p* < 0.001, ^**^*p* < 0.01, ^*^*p* < 0.1. Coef: mean estimated coefficient; SE: standard error; SD: standard deviation, indicating preference heterogeneity; CI: confidence interval (delta method); AIC: Akaike Information Criterion; BIC: Bayesian Information Criterion. Since monthly income was treated as a continuous variable, its estimated coefficient was less than 0.001.

**Table 8** Estimation of mixed logit model for job preferences (n = 875)

| Attribute level | Coef (SE) | SD (SE) | WTP (95%CI) |
| --- | --- | --- | --- |
| ASC (opt-out) | 9.530*** (0.271) | 2.477*** (0.116) |  |
| **Monthly income** | 0.0005454*** (0.0000162) | 0.0001366*** (0.000013) |  |
| **Work location: village or township (ref)** |  |  |  |
| county | 1.009*** (0.073) | 0.752*** (0.105) | 1849 (1601, 2094) |
| city | 2.329*** (0.093) | 1.369*** (0.083) | 4270 (3986, 4599) |
| **Work environment: poor (ref)** |  |  |  |
| common | 1.213*** (0.074) | 0.778*** (0.092) | 2224 (1955, 2520) |
| excellent | 1.669*** (0.077) | 0.564*** (0.118) | 3060 (2808, 3354) |
| **Training and career development opportunities: insufficient (ref)** |  |  |  |
| general | 0.445***(0.064) | 0.037 (0.164) | 815 (601, 1041) |
| sufficient | 1.362*** (0.080) | 1.171*** (0.078) | 2497 (2233, 2785) |
| **Workload: 60 h/week (ref)** |  |  |  |
| 50 h/week | 0.442*** (0.062) | 0.100 (0.167) | 811 (588, 1021) |
| 40 h/week | 0.934*** (0.067) | 0.784*** (0.085) | 1713 (1475, 1959) |
| **Professional recognition: low (ref)** |  |  |  |
| normal | 0.850*** (0.066) | 0.124 (0.157) | 1559 (1332, 1782) |
| high | 1.165*** (0.071) | 0.739*** (0.090) | 2135 (1889, 2397) |
| N | 875 | | |
| Observation | 31371 | | |
| Log likelihood | −7161.9907 | | |
| LR χ^2^ | 3118.20 | | |
| Prob > χ^2^ | < 0.0001 | | |

^***^*p* < 0.001, ^**^*p* < 0.01, ^*^*p* < 0.1. Coef: mean estimated coefficient; SE: standard error; SD: standard deviation, indicating preference heterogeneity; CI: confidence interval (Krinsky‐Robb parametric bootstrap). Since monthly income was treated as a continuous variable, its estimated coefficient was less than 0.001.

**Fig. 2.** The acceptance probability of rural/urban position under simulated incentive packages. Labels 1–13 on the horizontal axis represent 13 incentive packages respectively: Baseline (1), 40 h/week (2), high professional recognition (3), sufficient training and career development opportunities (4), 9000 CNY/month (5), excellent work environment (6), sufficient training and career development opportunities + high professional recognition (7), 9000 CNY/month + sufficient training and career development opportunities (8), excellent work environment + sufficient training and career development opportunities (9), 12,000 CNY/month (10), 9000 CNY/month + excellent work environment (11), excellent work environment + sufficient training and career development opportunities + high professional recognition (12), 9000 CNY/month + excellent work environment + sufficient training and career development opportunity (13).

**Table 9** Estimation of mixed logit model for altruistic preferences

| Attribute | Coef (SE) | SD (SE) | MRS (95%CI) |
| --- | --- | --- | --- |
| Profit *π*(*q*) | 1.026*** (0.041) | 0.826*** (0.038) |  |
| Benefit *B*(*q*) | 1.138*** (0.032) | 0.719*** (0.028) | 1.109 (1.032, 1.197) |
| N | 875 | | |
| Observation | 86625 | | |
| Log likelihood | −11980.383 | | |
| LR χ^2^ | 4605.76 | | |
| Prob > χ^2^ | < 0.0001 | | |

^***^*p* < 0.001, ^**^*p* < 0.01, ^*^*p* < 0.1. Coef: mean estimated coefficient; SE: standard error; SD: standard deviation, indicating preference heterogeneity; CI: confidence interval (Krinsky‐Robb parametric bootstrap).

**Table 10** Estimation of mixed logit model for job preferences in low-altruism group

| Attribute level | Coef (SE) | SD (SE) | WTP (95%CI) |
| --- | --- | --- | --- |
| ASC (opt-out) | 9.224*** (0.884) | 3.090*** (0.340) |  |
| **Monthly income** | 0.0006338*** (0.0000569) | 0.0001423*** (0.0000328) |  |
| **Work location: village or township (ref)** |  |  |  |
| county | 1.124*** (0.165) | 0.402 (0.330) | 1773 (1316, 2235) |
| city | 2.547*** (0.288) | 1.491*** (0.202) | 4019 (3438, 4681) |
| **Work environment: poor (ref)** |  |  |  |
| common | 1.160*** (0.173) | 0.862** (0.250) | 1831 (1332, 2438) |
| excellent | 1.367*** (0.190) | 0.594 (0.372) | 2158 (1687, 2699) |
| **Training and career development opportunities: insufficient (ref)** |  |  |  |
| general | 0.447**(0.159) | 0.265 (0.460) | 705 (247, 1165) |
| sufficient | 1.146*** (0.229) | 1.193*** (0.193) | 1808 (1184, 2395) |
| **Workload: 60 h/week (ref)** |  |  |  |
| 50 h/week | 0.144 (0.147) | 0.440 (0.272) | 228 (−238, 664) |
| 40 h/week | 0.467** (0.151) | 0.733** (0.217) | 736 (278, 1235) |
| **Professional recognition: low (ref)** |  |  |  |
| normal | 0.854*** (0.165) | 0.512 (0.317) | 1347 (888, 1814) |
| high | 1.049*** (0.179) | 0.956*** (0.203) | 1654 (1132, 2206) |
| N | 170 | | |
| Observation | 6099 | | |
| Log likelihood | −1344.7269 | | |
| LR χ^2^ | 753.24 | | |
| Prob > χ^2^ | < 0.0001 | | |

^***^*p* < 0.001, ^**^*p* < 0.01, ^*^*p* < 0.1. Coef: mean estimated coefficient; SE: standard error; SD: standard deviation, indicating preference heterogeneity; CI: confidence interval (Krinsky‐Robb parametric bootstrap). Since monthly income was treated as a continuous variable, its estimated coefficient was less than 0.001.

**Table 11** Estimation of mixed logit model for job preferences in medium-altruism group

| Attribute level | Coef (SE) | SD (SE) | WTP (95%CI) |
| --- | --- | --- | --- |
| ASC (opt-out) | 10.313*** (0.463) | 2.074*** (0.162) |  |
| **Monthly income** | 0.0005929*** (0.0000277) | 0.0001178*** (0.0000197) |  |
| **Work location: village or township (ref)** |  |  |  |
| county | 1.203*** (0.119) | 0.766*** (0.164) | 2028 (1660, 2387) |
| city | 2.536*** (0.149) | 1.251*** (0.133) | 4277 (3878, 4740) |
| **Work environment: poor (ref)** |  |  |  |
| common | 1.421*** (0.121) | 0.696*** (0.166) | 2396 (1998, 2850) |
| excellent | 1.910*** (0.129) | 0.648*** (0.167) | 3221 (2834, 3676) |
| **Training and career development opportunities: insufficient (ref)** |  |  |  |
| general | 0.504*** (0.106) | 0.293 (0.228) | 850 (522, 1196) |
| sufficient | 1.635*** (0.137) | 1.331*** (0.132) | 2758 (2348, 3205) |
| **Workload: 60 h/week (ref)** |  |  |  |
| 50 h/week | 0.486*** (0.101) | 0.299 (0.216) | 820 (483, 1131) |
| 40 h/week | 1.000*** (0.106) | 0.770*** (0.134) | 1687 (1343, 2052) |
| **Professional recognition: low (ref)** |  |  |  |
| normal | 0.844*** (0.107) | 0.293* (0.169) | 1423 (1088, 1755) |
| high | 1.077*** (0.115) | 0.807*** (0.126) | 1816 (1447, 2205) |
| N | 353 | | |
| Observation | 12642 | | |
| Log likelihood | −2884.5452 | | |
| LR χ^2^ | 1046.71 | | |
| Prob > χ^2^ | < 0.0001 | | |

^***^*p* < 0.001, ^**^*p* < 0.01, ^*^*p* < 0.1. Coef: mean estimated coefficient; SE: standard error; SD: standard deviation, indicating preference heterogeneity; CI: confidence interval (Krinsky‐Robb parametric bootstrap). Since monthly income was treated as a continuous variable, its estimated coefficient was less than 0.001.

**Table 12** Estimation of mixed logit model for job preferences in high-altruism group

| Attribute level | Coef (SE) | SD (SE) | WTP (95%CI) |
| --- | --- | --- | --- |
| ASC (opt-out) | 10.944*** (0.732) | 2.738*** (0.291) |  |
| **Monthly income** | 0.0005811*** (0.0000421) | 0.0001813*** (0.000029) |  |
| **Work location: village or township (ref)** |  |  |  |
| county | 0.821*** (0.201) | 1.338*** (0.226) | 1413 (738, 2057) |
| city | 2.823*** (0.252) | 1.817*** (0.230) | 4857 (4142, 5724) |
| **Work environment: poor (ref)** |  |  |  |
| common | 1.594*** (0.195) | 0.902*** (0.191) | 2743 (2109, 3513) |
| excellent | 2.145*** (0.199) | 0.355 (0.320) | 3691 (3099, 4438) |
| **Training and career development opportunities: insufficient (ref)** |  |  |  |
| general | 0.868*** (0.170) | 0.134 (0.282) | 1494 (958, 2044) |
| sufficient | 2.230*** (0.212) | 0.973*** (0.188) | 3838 (3207, 4545) |
| **Workload: 60 h/week (ref)** |  |  |  |
| 50 h/week | 0.476** (0.153) | 0.139 (0.290) | 819 (298, 1311) |
| 40 h/week | 1.090*** (0.167) | 0.821*** (0.205) | 1875 (1317, 2488) |
| **Professional recognition: low (ref)** |  |  |  |
| normal | 0.735*** (0.161) | 0.063 (0.237) | 1265 (734, 1798) |
| high | 1.256*** (0.179) | 0.842*** (0.212) | 2162 (1574, 2796) |
| N | 172 | | |
| Observation | 6165 | | |
| Log likelihood | −1317.2566 | | |
| LR χ^2^ | 669.74 | | |
| Prob > χ^2^ | < 0.0001 | | |

^***^*p* < 0.001, ^**^*p* < 0.01, ^*^*p* < 0.1. Coef: mean estimated coefficient; SE: standard error; SD: standard deviation, indicating preference heterogeneity; CI: confidence interval (Krinsky‐Robb parametric bootstrap). Since monthly income was treated as a continuous variable, its estimated coefficient was less than 0.001.

**Fig. 3.** The cumulative frequency distribution graph of marginal rate of substitution (MRS). It should be noted that the estimated MRS greater than 14 at the individual level were treated as 14, because the increase in patient health benefit caused by the loss of individual profit theoretically cannot exceed the maximum value of patient health benefit set at 14 in this study.
